# Stereotype threat effects on deaf and hard-of-hearing college students’ mathematics performance

**DOI:** 10.1093/jdsade/enaf088

**Published:** 2026-01-28

**Authors:** Ronald R Kelly, Peter C Hauser, Gerald P Berent, Susan Rizzo, Jessica Contreras, Jeremy P Jamieson

**Affiliations:** National Technical Institute for the Deaf, Rochester Institute of Technology, Rochester, NY, United States; National Technical Institute for the Deaf, Rochester Institute of Technology, Rochester, NY, United States; National Technical Institute for the Deaf, Rochester Institute of Technology, Rochester, NY, United States; National Technical Institute for the Deaf, Rochester Institute of Technology, Rochester, NY, United States; Department of Leadership, Research, & Policy, University of Colorado Colorado Springs (UCCS), Colorado Springs, CO, United States; Department of Psychology, University of Rochester, Rochester, NY, United States

## Abstract

This study examined whether stereotype threat degrades deaf and hard-of-hearing (DHH) college students’ math performance. The DHH participants self-assigned their social identity as “deaf” or “hard of hearing” irrespective of audiological assessment. Social identity is central to experiencing stereotype threat and being deaf or hard of hearing may activate negative biases which trigger a stereotype threat that impacts test performance. A sample of college students (216 deaf, 128 hard of hearing, 152 hearing) were randomly assigned to either a stereotype threat or no-threat test condition and tested on arithmetic, modular arithmetic, and quantitative Graduate Record Examination–type math problems. The deaf and hard-of-hearing participants tested under the stereotype threat condition underperformed compared to those under the no-threat condition. Further exploratory analyses demonstrated that female participants underperformed male participants and African American/Black DHH participants underperformed White DHH participants. This double-threat jeopardy finding of multiple marginalized identities is consistent with the minority stress model. Overall, results are consistent with previous research in which a social identity is linked to a negative stereotype and both the stereotype and linked identity impact performance. This study demonstrated that *deaf* and *hard of hearing* are social constructs, and the results provide empirical support for the social model of disability.

The medicalization of deaf and hard-of-hearing (DHH) individuals as disabled people can have psychologically destructive effects. Here, we argue that deafness, per se, is a social construct like race, class, and gender as opposed to the medical model that views deafness as a problem with a person’s body. Sociologists and disability studies scholars support the social model that disability is a societal problem where there are social barriers and social attitudes toward different types of bodies (Mauldin, 2020; Mitra, 2006; Shakespeare, 2010). Recent studies have demonstrated that DHH individuals experience *audism*, which is negative biases against the deaf community (Eckert & Rowley, 2013; Hauser et al., 2010; Kurz et al., 2016) and some internalized audism which negatively affects their mental health (Tomaszewski et al., 2025). The Minority Stress Theory (Meyer, 2003) of how individuals from stigmatized social categories are exposed to excess stress can explain the effect of audism on mental health as found in many other stigmatized groups (see Hoy-Ellis, 2023, for review). The present study focused on a specific phenomenon according to which perceived oppressive stressors can cause anxiety and then interfere with individuals’ success. Specifically, the study is a classic social psychology experiment that provides empirical evidence supporting a social model where DHH peoples’ fear of confirming negative stereotypes about their social identity, but not their perceived medical condition, can interrupt their academic performance, specifically mathematics performance. While the discipline of Deaf Studies has discussed the social model, including audism biases, microaggressions, and oppression experienced by the DHH community (e.g., Leigh, 2020; O’Connell, 2022; Tomaszewski et al., 2025), this paper presents the first experimental study which illustrates the effects of *stereotype threat* on young DHH adults enrolled in university baccalaureate programs.

## Stereotype threat

*Stereotype threat* is a widely studied social psychological construct that refers to the concern individuals feel when at risk of confirming, as self-characteristic, a negative stereotype about their group (Inzlicht & Schmader, 2012; Steele & Aronson, 1995). These performance concerns tied to negative stereotypes can undermine test performance. The classical experiment typically involves an experimental group of privileged and marginalized individuals taking a test where the instructions explicitly state that the marginalized group generally does not perform as well as the privileged group. The control group of both privileged and marginalized individuals do not receive the stereotype threat in the instructions. Privileged and marginalized individuals in the control group do not show differences in test performance, but in the experimental group the marginalized group underperforms compared to the privileged group. It is believed that the marginalized group experiences a fear of confirming the untrue stereotype about their group and the fear causes test anxiety that interferes with test performance, hence a perceived stereotype threat. The threat of confirming a stereotype specific to the knowledge, skills, or abilities being evaluated has consistently been shown to contribute to the underperformance of individuals who are members of stigmatized groups.

Over the past decade, there have been hundreds of stereotype threat studies validating the classical research method for supporting the social constructs of race, gender, and marginalization. For example, research has documented the negative impacts of stereotype threat on minority and low socioeconomic status individuals’ performance on standardized tests (Spencer & Castano, 2007; Steele & Aronson, 1995), women’s mathematics performance (Jamieson & Harkins, 2009; Spencer et al., 1999) and sport activities (Gentile et al., 2018), and White athletes’ athletic performance (Stone, 2002; Stone et al., 1999). More recently, Drače et al. (2025) examined the intersection of stereotype threat with emotions, showing a decrease in task performance under fear-based stereotype threat but not under anger-based threat. Steele (2010) contended that societal stereotypes are pervasive phenomena and that “negative stereotypes about our identities hover in the air around us” (p. 209). That is, stereotype threats are *always in the ether* and in our consciousness and thus have the potential to affect a wide array of domains and stigmatized groups. Individuals can also be susceptible to multiple negative stereotype threats in an evaluative situation. Gonzales et al. (2002) showed that gender-based, ethnicity-based, and race-based stereotype threat effects influenced Latino women’s performance on mathematical and spatial ability tests. Seo and Lee (2021) examined how stereotype threat in high school linked to teachers’ fixed mindsets about students resulted in Black and Latino males and White female students experiencing greater stereotype threat and anxiety adversely impacting achievement in mathematics classrooms.

For stereotype threat to affect an individual’s performance, the individual (a) must believe that the stereotype is applicable to his or her social identity, (b) must possess the requisite knowledge and skills to perform the task, and (c) his or her performance must be subject to evaluation (Jamieson & Harkins, 2011; Quinn et al., 2010; Wout et al., 2008). For example, a stereotype threat effect degrading performance on a math test could occur in an individual who is a member of a group stigmatized as possessing low math abilities and who is aware of that stigma but otherwise has the knowledge and skills to perform successfully on the math test. That is, stereotype threat is an *underperformance* effect.

## Social identity

Social identity and stigma are integral to the experience of stereotype threat: “Stereotype threat occurs when an identity is linked to a negative stereotype and the identity and stereotype are relevant in a particular situation” (Quinn et al., 2010, p. 382). Identity is a key factor influencing susceptibility to stereotype threat. Moreover, individual differences in identity can variably influence test performance. For example, Brown and Pinel (2003) showed that women who are normally high in stigma consciousness scored worse on a math test compared to women who are normally low in stigma consciousness (in the absence of any stereotype threat activation). Similarly, Schmader (2002) documented that women with higher levels of gender identification performed worse than men on a math test but women with lower levels of gender identification performed equivalently to men. Identity salience and stereotype threat also apply beyond gender, ethnicity, and race. Yopyk and Prentice (2005) showed that student athletes primed on their “athlete identity” performed less well on a math test than those primed on their “student identity.” While gender, ethnicity, and race identity threat to mathematics and other academic performance has been extensively examined, stereotype threat predicated on physical disability has not (for an exception, see May & Stone, 2014). The dearth of research in this area is notable given that stereotype threat elicits lower levels of self-integrity and well-being in blind individuals (Silverman & Cohen, 2014) and that university students with physical disabilities performed lower on a logic test when they knew that the evaluators were other students without disabilities compared to when they were evaluated by students with disabilities (Desombre et al., 2017). Moreover, stereotype threat effects may be particularly impactful in disabled populations because of the prevalence of negative stereotypes ascribed to these groups. For instance, students with physical disabilities are commonly perceived as incompetent, unproductive, dependent (Rohmer & Louvet, 2009), and intellectually challenged (Rohmer & Louvet, 2011).

## Deaf and hard-of-hearing students

Notably, no research to date has examined the impact of stereotype threat on DHH students, an historically stigmatized group that faces serious academic and test performance challenges. The present study addresses this research gap by testing effects of stereotype threat on self-identified deaf and hard-of-hearing college students’ assessed knowledge and skills in mathematics, a domain that is critical to their academic success, especially in studies leading to Science, Technology, Engineering, and Mathematics (STEM) careers. It is important for this gap to be addressed because many educators continue to believe that any academic performance discrepancies can be explained by the medical model without any realization how social factors can have an effect on learning and test performance. The extent to which deaf and hard-of-hearing students are vulnerable to stereotype threat effects may be tied to identity processes. Leigh (2009) noted that “labels are a form of stereotyping based on one’s perceived identification or similarity between the self and members of a specific category or group as contrasted with perceived differences between the self and outside group members” (p. 9). For example, the generally accepted definition of “hard of hearing” is “less deaf” (Wilson, 2001). Yet, some profoundly deaf people utilize spoken language for communication. When they do, they are often labeled “hard of hearing” despite their decibel-level profound hearing loss (Punch et al., 2006). For deaf and hard-of-hearing individuals, self-identifying their social identity often has nothing to do with objective audiological assessment but rather “… indicates various choices about self-representations, communication and language choices, individual functioning, and socialization with hearing and/or deaf persons” (Leigh, 2009, p. 9) in educational environments and in society in general. Because stereotype threat is activated only when an individual identifies with a negatively stereotyped group, the identity aspect of DHH individuals is central to this stereotype threat study.

With respect to DHH students’ mathematical knowledge and performance, research has shown that approximately half of DHH students at age 17 perform well below grade level (Qi & Mitchell, 2012). As a result, many DHH students enter college insufficiently prepared in mathematics (Kelly, 2008) and in math word problem solving (Blatto-Vallee et al., 2007). Furthermore, 55% of DHH students entering college rank below the 50th percentile in confidence in their mathematical and science abilities with another 31% ranking only between the 51st and 75th percentile (Albertini et al., 2012). In this context, the present study focused on DHH college students enrolled in 4-year bachelor’s degree programs because they are generally better prepared in mathematics than DHH students enrolled in 2-year associate degree programs. The rationale is, as noted above, that the detection of stereotype threat effects among members of a stigmatized group requires that they actually possess the knowledge and skills to perform successfully on the targeted assessment.

## Current research

The research explored in this study is guided by stereotype threat theory and mathematical research on DHH college students. The study assessed performance of DHH individuals enrolled in baccalaureate programs and a comparison group of hearing baccalaureate-level students. The deaf, hard-of-hearing, and hearing students were randomly assigned to one of two experimental conditions: (a) a “stereotype threat” test condition, in which task instructions activate the negative stereotype that *hearing students perform better than deaf and hard-of-hearing students on math tests*, and (b) a “no-threat” test condition, in which task instructions maintain that *hearing students do not perform any better* than deaf and hard-of-hearing *students on math tests*. In view of the discussion above, DHH participants were grouped on the basis of their own self-assigned social identities as “deaf” or “hard of hearing.” The primary research focus for this study is whether the math test performance of the self-assigned social identities of deaf and hard-of-hearing participants randomly assigned to the stereotype threat test condition differs from the performance of those randomly assigned to the no-threat test condition. This research question will be addressed by analyzing the following:

1. Group means for performance on the three mathematics tests and overall performance on the combined total score of the three mathematics tests;
2. Responses to the post-task experimental manipulation check questions on whether the hearing participants compared to the deaf and hard-of-hearing participants would differ on these math tests, including the assumption of higher performance by the hearing participants;
3. Perceptions of their test experience in terms of difficulty, stressfulness, and effort given to solving the math problems.

The implied null hypothesis is that there will be no difference in performance on the three math tests between the deaf and hard-of-hearing participants under the stereotype threat test condition and the deaf and hard-of-hearing participants under the no-threat test condition. The alternative research hypothesis is that the deaf and hard-of-hearing participants tested under the stereotype threat test condition will significantly underperform on the math tests relative to the deaf and hard-of-hearing participants under the no-threat test condition. If the results support the alternative research hypothesis, then the responses of the deaf and hard-of-hearing stereotype threat participants to the post-task experimental manipulation check questions and their perceptions of the test’s difficulty and stressfulness should be consistent with their underperformance and significantly different from the responses and perceptions of the deaf and hard-of-hearing participants tested under the no-threat test condition.

As noted above, negative stereotypes are pervasive (Steele, 2010) and people can be susceptible to multiple stereotype threats (Gonzales et al., 2002). The deaf and hard-of-hearing participants may be susceptible to multiple negative stereotype threats due to their gender, ethnicity, and/or race in addition to the primary experimental threat manipulation that was meant to activate stereotype threat relating to deaf and hard-of-hearing students’ underperformance in math. Sample size and power requirements were sufficient in all three groups (deaf, hard of hearing, and hearing) to further explore gender stereotype threat effects on math performance. There are minimally adequate participant numbers to explore impacts of stereotype threat relating to ethnicity and race (African American/Black, Hispanic/Latino, Asian, and White) but only among the combined DHH participants. However, the power requirements for ethnicity and race are not sufficient in a number of analysis cells to detect significance if it exists, which render nonsignificant findings inconclusive. The exploratory examination of gender, ethnicity, and race on performance was addressed by analyzing group means for overall performance on the combined total score for three mathematics tests.

## Method

The design of this research study consisted of three college participant groups: deaf, hard-of-hearing, and hearing students. The DHH participants self-assigned their social identity as deaf or hard of hearing irrespective of their audiological assessments. Within each group, participants were randomly assigned to either a stereotype threat test condition or a no-threat test condition. The analyses examined the three group performance differences between the stereotype threat test condition and the no-threat test condition, as well as gender differences. Ethnicity/racial differences were examined only with the combined DHH participants due to sample size limitations. Following are details of the methodology and procedures used in conducting this research study.

### Participants

To increase the probability that the deaf and hard-of-hearing college student participants possessed the ability to successfully solve the types of math problems administered in this research study, recruitment targeted only deaf and hard-of-hearing students who were enrolled in 4-year baccalaureate degree programs. Prior to recruiting students, a power analysis was conducted to determine the minimum sample size for each comparison group to detect a medium effect size of the experimental manipulation if it exists. Cohen’s (1977) power tables were utilized for *F* tests on means in the analysis of variance. For the primary group comparisons among the deaf, hard-of-hearing, and hearing participants, sample sizes between 60 and 100 provide power of .77 to .97 for detecting a medium effect of the experimental manipulation. These minimum sample size targets provided the basis for recruitment of participants. A total of 496 college students were recruited (216 self-assigned social identity as deaf, 128 self-assigned social identity as hard-of-hearing, and 152 were hearing), and their subsequent random assignment to the stereotype threat or no-threat experimental test conditions met or exceeded these minimum sample size targets.

The mean ages of the three participant groups were deaf, 22.09, *SD* = 3.19, hard of hearing, 21.10, *SD* = 3.3, and hearing, 21.97, *SD* = 3.00. For the participants randomly assigned to the two experimental conditions, there was no significant difference in mean age, *t* = 1.66, *p* = .10, between the stereotype threat condition, 22.03, *SD* = 3.28, and the no-threat condition, 21.56, *SD* = 3.03. The gender distribution of participating deaf and hard-of-hearing female (52%) and male (48%) participants approximated the gender distribution of all DHH students enrolled at the university, which is near 50:50 annually. This ratio contrasts with the university’s hearing student population, which is typically a 65:35 male-to-female ratio annually. Random assignment of the deaf and hard-of-hearing students to the stereotype threat and no-threat test conditions resulted in an approximately equal distribution by gender (female 89:90, male 86:79). A Pearson chi-squared test revealed no significant difference in randomly assigned gender of the deaf and hard-of-hearing participants to the two experimental conditions, χ^2^ = .19, *df* = 1, *p* = .67. The gender distribution for the participating hearing students, female (47%) and male (53%), randomly assigned to the two experimental conditions also resulted in an approximately equal distribution by gender (female 34:37, male 41:40). A Pearson chi-squared test showed no significant difference in randomly assigned gender of hearing participants to the two experimental conditions, χ^2^ = .11, *df* = 1, *p* = .74. The ethnicity and race distribution of the DHH participants approximated a similar distribution to the hearing participants (see Table 1), although the DHH sample was slightly more diverse. However, the hearing participant group did not have enough diversity in each ethnicity and race group for comparisons with the DHH group.

**Table 1:** Percentage distribution of DHH and hearing participants by ethnicity and racial background.

Ethnicity and race	DHH percentage (*n* = 344)	Hearing percentage (*n* = 152)
White	44.3	60.0
Asian African American/Black	20.1 16.0	20.4 3.9
Hispanic/Latino	9.3	13.2
Mixed/Multi-background Indigenous or Alaska Native Hawaiian or other Pacific Other	6.9 .01 .01 3.4	0 0 0 2.5
Total percentage	100%	100%

## Materials

### Assessment measures

#### Pre-task assessments

There were five sets of pre-task assessments that participants completed in the following order prior to starting the three timed math tests:

##### Positive and Negative Affect Schedule Scale (Watson et al., 1988)

The Positive and Negative Affect Schedule Scale (PANAS) measures positive and negative affect at the time of the test. Participants rate 20 different words for their degree of positiveness on a 5-point Likert scale from 1 “not at all” to 5 “extremely.” Positive feelings and emotions are reflected in the words *interested*, *alert*, *excited*, *inspired*, *strong*, *determined*, *attentive*, *enthusiastic*, *active*, *proud*. Negative feelings and emotions are reflected in the words *irritable*, *distressed*, *ashamed*, *upset*, *nervous*, *guilty*, *scared*, *hostile*, *jittery*, *afraid*.

##### Abbreviated Math Anxiety Scale (Hopko et al., 2003)

The Abbreviated Math Anxiety Scale (AMAS) measures how anxious one feels about math on a 5-point Likert scale from 1 “low anxiety” to 5 “high anxiety.” The AMAS measures two types of math anxiety: (a) for learning math (using math tables, watching teacher work an algebraic equation, listening to a math lecture, listening to another student explain a math formula, starting a new chapter in a math book) and (b) for math evaluation (thinking about an upcoming math test the next day, taking a math examination, being given a difficult math homework assignment due the next class meeting, being given a pop quiz).

##### Stress Appraisal (Jamieson et al., 2016)

The Stress Appraisal measure consisted of four items measured on a 7-point Likert scale from 1 “strongly disagree” to 4 “neutral” to 7 “strongly agree.” Two items are positive: “I have the knowledge/ability to perform well on this type of exam”; “I’m the kind of person that does well in these types of situations.” Two items are negative: “The upcoming exam is very demanding”; “It will take a lot of effort to complete.”

##### Implicit Theories of Intelligence Measure (Levy et al., 1998)

The Implicit Theories of Intelligence Measure uses a 6-point Likert scale from 1 “strongly disagree” to 6 “strongly agree” to assess whether one agrees that one’s intelligence is set and unchangeable or that it is mutable. Three items convey that intelligence is unchangeable: “You have a certain amount of intelligence and can’t do much to change it”; “Your intelligence is something about you that you can’t change very much”; “You can learn new things but can’t really change your basic intelligence.” Three items convey that intelligence can be changed: “No matter who you are, you can change your intelligence a lot”; “You can always greatly change how intelligent you are”; and “No matter how much intelligence you have, you can always change it quite a bit.”

##### Achievement Goals Measure (Elliot & Murayama, 2008)

The Achievement Goals Measure consists of 12 items divided into four subscales: (1) mastery-approach (aim to completely master the course materials, learn as much as possible, and strive to thoroughly understand content); (2) mastery-avoidance (avoid learning less than I possibly could, strive to avoid an incomplete understanding of class materials, and avoid learning less than is possible); (3) performance-approach (aim to perform well relative to other students, strive to do well compared to other students, and aim to perform better than other students); and (4) performance-avoidance (aim to avoid doing worse than other students, strive to avoid performing worse than others, and avoid performing poorly compared to others). A 5-point Likert scale was used to measure responses to the 12 items, from 1 “strongly disagree” to 5 “strongly agree.”

### Math test materials

The three sets of timed math tests consisted of simple arithmetic operations, modular arithmetic, and Graduate Record Examination (GRE)–type problems.

#### Simple arithmetic operations (add, subtract, divide, multiply)

There were 144 problems in a 5-min timed test on which students were to complete as many problems as possible. All problems were presented in a vertical orientation reflecting the traditional school approach taught in the U.S. educational system. Carry operations were randomized within blocks to ensure similar difficulty of items across participants. See Appendix for example of the arithmetic problem format.

#### Modular arithmetic

There were 50 problems in a 10-min timed test in which students were to complete as many problems as possible. Modular problems used novel symbols to represent arithmetic operations, where “Ξ” indicates subtract and “mod” indicates divide. To be “True,” the mod number must divide evenly and not result in any decimals in the answer. See Appendix for example of the modular arithmetic problem format.

#### Graduate Record Examinations “GRE-Type”

There were 30 problems in a 20-min timed test in which students were to complete as many as possible. The problems were modeled after the GRE (Educational Testing Services, 2024). See Appendix for two example formats of the GRE-type multiple-choice problems.

### Post-task questions

Upon completion of the three timed sets of math problems (arithmetic, modular, and GRE-type), each participant ranked the following post-task questions using a 7-point Likert scale. Items 1–2 are checks on the stereotype threat and non-threat experimental manipulation. Items 4–6 are test experience items. Item 3 was included to check whether the students were paying attention.

1. To what extent are there differences between hearing and deaf/hard-of-hearing people on these math tasks?
2. How well do you think hearing students performed on these math tasks?
3. If you are paying attention, please click “5” on the rating scale.
4. How difficult were the problems on these math tasks?
5. How stressful were the problems on these math tasks?
6. How much effort did you give to solving the problems on these math tasks?

### Procedure

Students were recruited during 2016–2017 via email and scheduled for individual 1-hr testing sessions. Randomization was used at three levels to prevent bias in assignment to the experimental conditions or order effects on the sequencing of problems and the three math sets. Each participant was tested individually and randomly assigned (1) to either the stereotype threat or no-threat test condition, and (2) to one of six sequences of math problem sets—Arithmetic > Modular Arithmetic > GRE-type Problems; Modular Arithmetic > GRE-type Problems > Arithmetic; GRE-type Problems > Arithmetic > Modular Arithmetic; etc. And (3) each problem set consisted of the same randomly sequenced math items.

Except for the consent form, the remainder of the testing session was conducted online via a computer program per the following sequence:

1. Background information including self-identification as (a) deaf, hard of hearing, or hearing, (b) female or male, (c) Hispanic or Latino, American Indian/Alaska Native, Asian, Black/African American, Native Hawaiian/Other Pacific Islander, White, mixed/multi-racial, other; and specification of (d) age (10 min)
2. Pre-task assessments as described above (10 min)
3. Video instructions (signed, spoken, and captioned) specific to each participant’s randomly assigned test condition (2 min). Following the classical stereotype threat experimental procedures that are replicated here, an overt trigger was used for both the stereotype threat and no-threat test conditions because it is direct and obvious, and cues either a negative threat or positive thought related to the test task. An overt reminder of the stereotype does not, of course, create the stereotype. However, it plausibly activates an extant internalized stereotype, potentially increasing anxiety and undermining test performance. All participants were urged to complete as many math problems as possible within the time limits allocated for each problem set and to concentrate and do their best. The following explicit threat and no-threat wordings were given, respectively, to participants assigned to the two test conditions:
  Stereotype threat condition: “Research has shown that hearing students perform better than deaf and hard-of-hearing students on math tests.”
  No-threat condition: “Research has shown that hearing students do not perform any better than deaf and hard-of-hearing students on math tests.”
4. Three sets of math problems: Arithmetic, 5 min; Modular Arithmetic, 10 min; and GRE-type Problems, 20 min. Problem sets were allocated a total of 35 min to complete.
5. Post-task questionnaire (5 min)

Upon completion of the post-task questionnaire, each participant was asked if they had any questions or personal concerns about their research experience. They were thanked, debriefed, and compensated for participating in the research study.

## Results

All hypotheses were tested at alpha level = .05. To control for multiple comparisons, all post hoc comparisons utilized a Bonferroni correction. Each DHH participant was asked to self-assign their social identify as “deaf” or “hard of hearing.” Of the 344 DHH participants, 327 had an audiogram on record. Of those, only 57 measured within the hearing loss decibel range for mild to moderate, whereas 270 measured in the decibel range for severe to profound hearing loss. Sixty-three percent of the 128 self-identified hard-of-hearing students (*n* = 78) had an audiogram that placed them in the severe to profound hearing loss decibel range, and 5.4% of the 216 self-identified deaf students (*n* = 11) had an audiogram that placed them in the moderate loss decibel range. A Pearson chi-squared test showed there was a significant difference between participants’ objectively measured decibel hearing loss ranges and their deaf and hard-of-hearing self-assigned social identities, χ^2^ = 53.67, *df* = 1, *p* = .0005.

No statistically significant differences existed between deaf and hard-of-hearing participants in their math and language college entry scores or in cumulative grade point average in their college courses. Table 2 shows the American College Test (ACT) mean scores for math, reading, English, and science, as well as their cumulative GPA. The ACT is a national standardized test designed to measure a high school student’s readiness for college-level coursework. Cumulative grade point average was calculated for all of the deaf and hard-of-hearing participants’ college courses completed up to the time of this research study.

**Table 2:** Comparison of deaf and hard-of-hearing participants’ math and language scores at entry to college and their cumulative grade point averages (GPA).

Assessment measures at entry to college	Deaf participants’ mean scores (*SD*)	HH participants’ mean scores (*SD*)	Significance (2-tailed *t*)	Mean difference
ACT Math	16.45 (9.7)	15.88 (11.2)	n.s.	.57
ACT Reading	15.79 (9.7)	16.29 (12.0)	n.s.	−.51
ACT English	13.77 (8.8)	14.38 (10.9)	n.s.	−.61
ACT Science	16.99 (9.8)	16.35 (11.5)	n.s.	.65
Cumulative GPA	2.55 (1.3)	2.32 (1.4)	n.s.	.23

Regarding the results of the pre-task assessments (see Table 3), five areas showed a significant difference between the three groups of deaf, hard-of-hearing, and hearing participants: PANAS Negative Feelings, Anxiety for Learning Math, Anxiety for Math Evaluation, Negative Stress Appraisal, and Implicit Theories for Intelligence Unchangeable. First, the PANAS Negative Feelings ratings showed a significant one-way ANOVA, *F*(2, 490) = 3.64, *p* = .027, with the multiple comparisons revealing that the hearing participants had a significantly lower rating for Negative Feelings than the deaf participants (*M*_diff_ = .13, *p* = .036, CI_95_ = [−.26; −.01]; *SE* = .05), but not significantly different than the hard-of-hearing participants (*M*_diff_ = −.05, *p* = .68, CI_95_ = [−.19; −.09]; *SE* = .05). Second, the Anxiety for Learning Math one-way ANOVA was also significant, *F*(2, 490) = 8.29, *p* = .001, and the multiple comparisons revealed that the hearing participants had significantly lower anxiety ratings than both the deaf participants (*M*_diff_ = −.30, *p* = .001, CI_95_ = [−.50; −.10]; *SE* = .08) and the hard-of-hearing participants (*M*_diff_ = −.31, *p* = .004, CI_95_ = [−.54; −.08]; *SE* = .09). Third, the analysis for the Math Evaluation Anxiety ratings was statistically significant, *F*(2, 490) = 4.15, *p* = .016, with the multiple comparisons revealing that the hearing participants had significantly lower anxiety than the hard-of-hearing participants (*M*_diff_ = −.35, *p* = .02, CI_95_ = [−.65; −.05]; *SE* = .12) but not the deaf participants (*M*_diff_ = −.10, *p* = .63, CI_95_ = [−.36; −.16]; *SE* = .11). Fourth, the one-way ANOVA for the Negative Stress Appraisal was also significant, *F*(2, 490) = 35.95, *p* = < .001 with the multiple comparisons showing that the hearing participants’ ratings for negative stress were significantly lower than both the deaf (*M*_diff_ = −1.21, *p* = <.001, CI_95_ = [−1.56; −.85]; *SE* = .14) and the hard-of-hearing participants’ ratings (*M*_diff_ = −.91, *p* = <.001, CI_95_ = [−1.31; −.51]; *SE* = .16). And fifth, the one-way analysis for Implicit Theories that intelligence is unchangeable was significant, *F*(2, 490) = 8.50, *p* = <.001, with the multiple comparisons revealing that the hearing participants had a statistically lower rating than the deaf participants (*M*_diff_ = −.55, *p* = .000, CI_95_ = [−.87; −.22]; *SE* = .13), but not the hard-of-hearing participants (*M*_diff_ = −.32, *p* = .10, CI_95_ = [−.69; −.05]; *SE* = .15). No other analyses for the pre-task assessments revealed any significant comparisons between the deaf, hard-of-hearing, and hearing participants.

**Table 3:** Five pre-task assessments: deaf, hard-of-hearing (HH), and hearing participants’ means, standard deviations, and significance.

Assessment	Deaf (*n* = 216) HH (*n* = 128) Hearing (*n* = 152)	Mean	*SD*	Significance One-way ANOVA
1. PANAS Positive	Deaf HH Hearing	3.14 3.10 3.16	.76 .81 .82	Not significant *F*(2, 490) = .29, *p* = .75
PANAS Negative	Deaf HH Hearing	1.50 1.42 1.37	.56 .45 .42	Significant *F*(2, 490) = 3.64, *p* = .027
2. Anxiety Learn Math	Deaf HH Hearing	1.83 1.84 1.53	.86 .79 .63	Significant *F*(2, 490) = 8.29, *p* = .001
Anxiety Math Evaluation	Deaf HH Hearing	3.09 3.33 2.98	1.02 1.06 .95	Significant *F*(2, 490) = 4.15, *p* = .016
3. Stress Appraisal Positive	Deaf HH Hearing	4.73 4.77 4.81	1.17 1.36 1.16	Not significant *F*(2, 490) = .16, *p* = .85
Negative	D HH Hearing	4.83 4.53 3.63	1.38 1.57 1.13	Significant *F*(2, 490) = 35.95, *p* = .000
4. Implicit Theories of Intelligence No change	Deaf HH Hearing	3.15 2.92 2.60	1.33 1.22 1.15	Significant *F*(2, 490) = 8.50, *p* = .000
Yes change	Deaf HH Hearing	4.40 4.48 4.29	1.10 1.04 1.16	Not significant *F*(2, 490) = 1.25, *p* = .29
5a. Achieve Mastery Approach	Deaf HH Hearing	4.27 4.24 4.21	.63 .74 .72	Not significant *F*(2, 490) = .34, *p* = .71
Avoidance	Deaf HH Hearing	3.55 3.51 3.71	1.02 1.02 .89	Not significant *F*(2, 490) = 1.93, *p* = .15
5b. Achieve Performance Approach	Deaf HH Hearing	3.83 3.98 3.91	.83 .77 .91	Not significant *F*(2, 490) = .24, *p* = .63
Avoidance	Deaf HH Hearing	3.78 3.87 3.75	1.03 1.07 1.14	Not significant *F*(2, 490) = .54, *p* = .58

In turning to the analysis of the math performance of the deaf, hard of hearing, and hearing students, it should first be noted that all three participant groups completed a similar number of problems in the time allocated for each of the three problem sets: Arithmetic, Modular Arithmetic, and GRE-Type math. Thus, the statistical analyses are conducted on similar amounts of data generated by each of the three participants groups. Table 4 presents the percentages of problems completed and correctly calculated for each of the three problem math sets.

**Table 4:** Mean percentage of math problems completed and correctly calculated per three math problem sets within the allocated time by the deaf, hard-of-hearing, and hearing participants.

	Deaf mean % (*SD*)	Hard of hearing mean % (*SD*)	Hearing mean % (*SD*)
Arithmetic (5 min)			
% Completed	.58 (.22)	.60 (.22)	.68 (.20)
% Correct	.55 (.22)	.58 (.22)	.66 (.20)
Modular arithmetic (10 min)			
% Completed	.90 (.17)	.89 (.18)	.97 (.09)
% Correct	.80 (.20)	.81 (.21)	.92 (.11)
GRE-Type Math (20 min)			
% Completed	.80 (.21)	.79 (.21)	.74 (.21)
% Correct	.30 (.14)	.31 (.16)	.44 (.20)

Second, the majors of the participants in each of the three groups represented a diversity of majors across all 11 colleges of the university. For the deaf participants, there were 75 different majors across the 11 colleges. The hard-of-hearing participants were represented by 51 different majors distributed across nine colleges. And the hearing participants had 72 different majors distributed across nine colleges. Table 5 provides the percentages of participants with majors in each college and the undecided status for each of the three participant groups.

**Table 5:** Mean percentage of majors across 11 colleges by deaf, hard-of-hearing, and hearing participants

Colleges	Deaf (*n* = 216) Mean %	Hard of hearing (*n* = 128) Mean %	Hearing (*n* = 152) Mean %
Art & Design	11.1	8.6	6.6
Business	22.2	11.0	22.0
Computing	19.3	25.0	9.2
Engineering	10.2	14.1	8.0
Engineering Technology	1.2	2.4	0
Health Sciences	5.0	9.3	6.3
Liberal Arts	13.0	10.2	2.5
NTID	2.3^a^	2.4^a^	12.4^b^
Science	6.5	14.0	31.0
Sustainability	1.0	0	0
Individualized Study	3.2	0	1.0
Undecided^c^	5.0	3.0	1.0
Total	100.0	100.0	100.0

^a^1st year Master’s degree DHH students in Teacher Education

^b^Baccalaureate degree students in interpreting and 1st year Master’s degree students

^c^A small number of students indicated “undecided” major without identifying a college

Initially, a mixed-model analysis of variance (between-within subjects) was performed to examine the impact of the two test conditions (stereotype threat, no-threat) on the percent-correct test scores of the three participant groups (deaf, hard of hearing, hearing) for the three different sets of math problems (arithmetic, modular, GRE-type). There were no significant interactions between performance on the three sets of math problems among the three participant groups, *F* (4, 980) = 1.79, *p* = .13. Likewise, there were no significant interactions between the three sets of math problems and the stereotype threat/no-threat conditions, *F*(2, 980) = 1.98, *p* = .14. Furthermore, no three-way interaction occurred between the three sets of math problems, the two participant groups, and the two test conditions, *F*(4, 980) = 2.04, *p* = .09. However, there was a robust main effect comparing the mean percent correct responses on the three sets of math problems, *F*(2, 980) = 1670.88, *p* < .001, η_p_^2^ = .77, a very large effect size showing that all participant groups performed best on the modular problems, less well on the arithmetic problems, and least well on the GRE-type problems.

Given that no interactions were significant and that the three groups performed consistently across the three math problem sets (arithmetic, modular, and GRE-type), all subsequent analyses were conducted on the combined total math score. Overall means were calculated for the three math problem sets by standardizing the raw correct scores for each individual to *z* scores and then averaging all the *z* scores from the three math problem sets for each participant.

**Figure 1:**
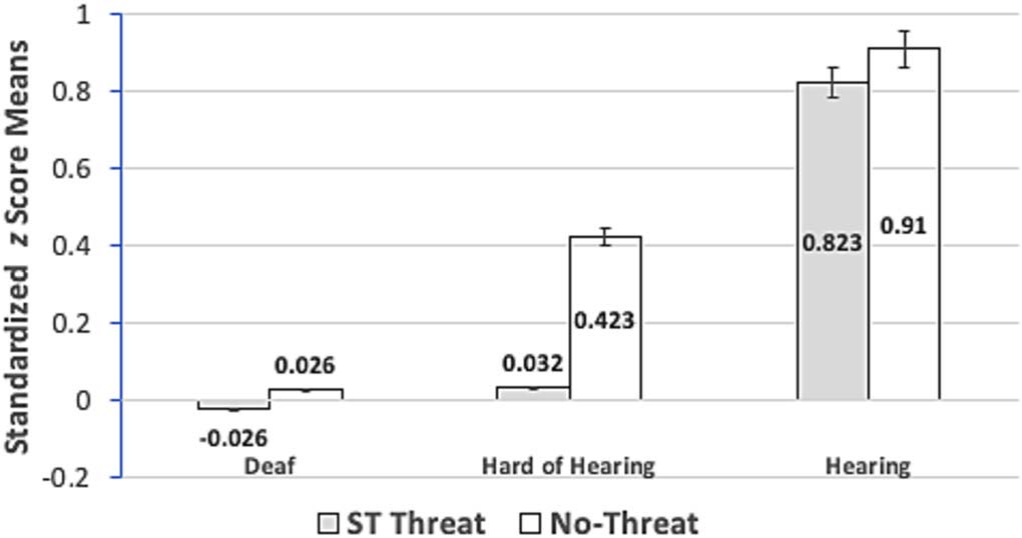
Deaf, hard of hearing, and hearing college students’ overall performance on the combined three math problem sets under stereotype threat (ST) and no-threat test conditions. Note. Standard errors are represented in the figure by the error bars for each column. Zero equals mean group performance. Positive and negative *z* score averages represent how far each group performed above or below their group mean. Each *z* score equals 1 *SD* ranging from −3 to +3.

To examine the main effect of whether the math test performance of the three participant groups was equivalent or different, a 3 (deaf, hard of hearing, hearing) × 2 (stereotype threat, no-threat) ANOVA was conducted on the overall total performance means based on each individual participant’s *z* score average, *F*(2, 490) = 45.34, *p* < .001, η_p_^2^ = .15, a large effect size. The main effect of stereotype threat versus no threat conditions was also significant, *F*(1, 490) = 4.88, *p* = .028, η_p_^2^ = .01, a small effect size. There was no interaction between the three participant groups and the threat/no-threat experimental condition, *F*(2, 490) = 1.68, *p* = .19. Post hoc comparisons revealed that the deaf group’s performance did not statistically differ from the hard-of-hearing group’s performance (*M*_diff_ = −.23, *p* = .059, CI_95_ = [−.53; .007]; *SE* = .097). However, the deaf group’s performance was significantly lower than the hearing group’s performance (*M*_diff_ = −.87, *p* < .001, CI_95_ = [−1.26; −.73]; *SE* = .092), Cohen’s *d* = −.98, a large effect size. Similarly, the hard-of-hearing group’s performance was significantly lower than the hearing group’s performance (*M*_diff_ = −.64, *p* < .001, CI_95_ = [−1.03; −.44]; *SE* = .104), Cohen’s *d* = −.74, a large effect size (see Figure 1).

**Figure 2:**
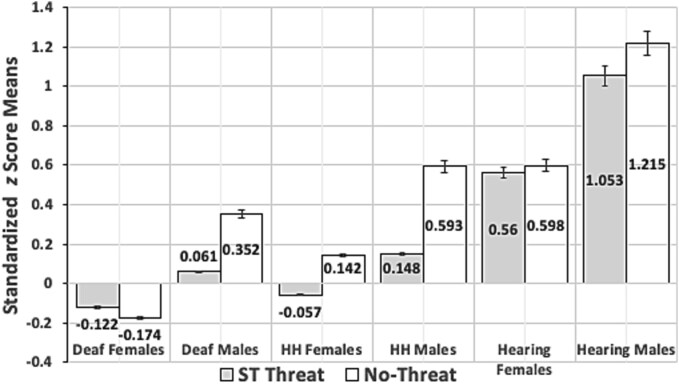
Gender influence on the deaf, hard of hearing, and hearing college students’ overall performance on the combined three math problem sets under stereotype threat (ST) and no-threat test conditions. Note. Standard errors are represented in the figure by the error bars for each column. Zero equals mean group performance. Positive and negative *z* score averages represent how far each group performed above or below their group mean. Each *z* score equals 1 *SD* ranging from −3 to +3.

To explore the gender intersectionality on the three groups’ overall math performance, a 3 (deaf, hard of hearing, hearing) × 2 (female, male) × 2 (stereotype threat, no-threat) ANOVA was conducted. The main effect of the three participant groups was significant, *F*(2, 484) = 43.75, *p* < .001, η_p_^2^ = .15, a large effect size. Post hoc comparisons showed that the deaf group’s performance did not statistically differ from the hard-of-hearing group’s performance (*M*_diff_ = −.17, *p* = .19, CI_95_ = [−.40; .05]; *SE* = .096). However, the hearing group performed significantly higher than both the deaf group (*M*_diff_ = −.83, *p* = .001, CI_95_ = [−1.04; −.62]; *SE* = .090), Cohen’s *d* = −.98, a large effect size, and the hard-of-hearing group (*M*_diff_ = −.65, *p* = .001, CI_95_ = [−.89; −.41]; *SE* = .103), Cohen’s *d* = −.77, a medium to near large effect size. The main effect of gender was also significant, *F*(1, 484) = 27.34, *p* < .001, η_p_^2^ = .053, a small effect size. The main effect of stereotype threat/no-threat condition was also significant, *F*(1, 490) = 5.25, *p* = .022, η_p_^2^ = .01, a small effect size. There were no significant interaction effects among the three participant groups, gender, and stereotype threat/no-threat conditions. As shown in Figure 2, female participants consistently underperformed relative to male participants in the deaf, hard-of-hearing, and hearing participant groups. Thus, being female and DHH activates a dual stereotype that threatens to degrade math performance with combined effect.

**Figure 3:**
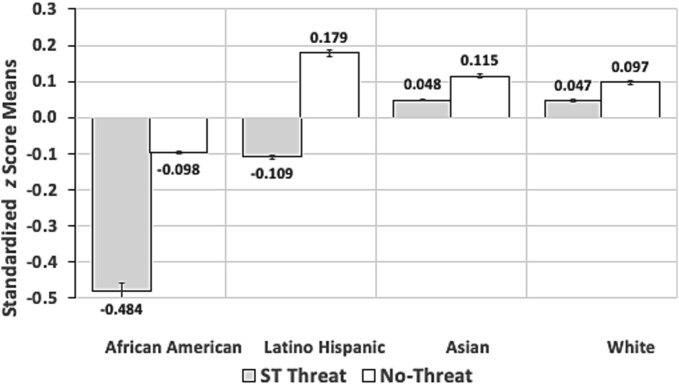
Ethnicity and race influence on deaf and hard-of-hearing college students’ overall performance on the combined three math problem sets under stereotype threat (ST) and no-threat test conditions. Note. Standard errors are represented in the figure by the error bars for each column. Zero equals mean group performance. Positive and negative *z* score averages represent how far each group performed above or below their group mean. Each *z* score equals 1 *SD* ranging from −3 to +3.

To explore the influence of ethnicity and race, only the deaf and hard-of-hearing participant groups combined, but not the hearing group, had enough participants to include the following four ethnic and racial groups: African American/Black, Latino/Hispanic, Asian, and White. A 4 (ethnicity and race groups) × 2 (stereotype threat, no-threat) ANOVA was conducted showing that the main effect of ethnicity and race was significant, *F*(3, 296) = 3.024, *p* = .030, η_p_^2^ = .03, a small effect size. The main effect of stereotype threat versus no-threat conditions was not significant, *F*(1, 296) = 3.16, *p* = .077. There was also no significant interaction between the three participant groups and the threat/no-threat experimental condition, *F*(3, 296) = .708, *p* = .55. Post hoc comparisons revealed that the African American/Black group’s performance was significantly lower than the White participant group’s performance (*M*_diff_ = −.363, *p* = .028, CI_95_ = [−.69; −.034]; *SE* = .13), Cohen’s *d* = −.45, a near-medium effect size. The post hoc comparisons between the African American/Black and the Asian and Latino/Hispanic participant groups were not significant and were inconclusive (see Figure 3).

**Figure 4:**
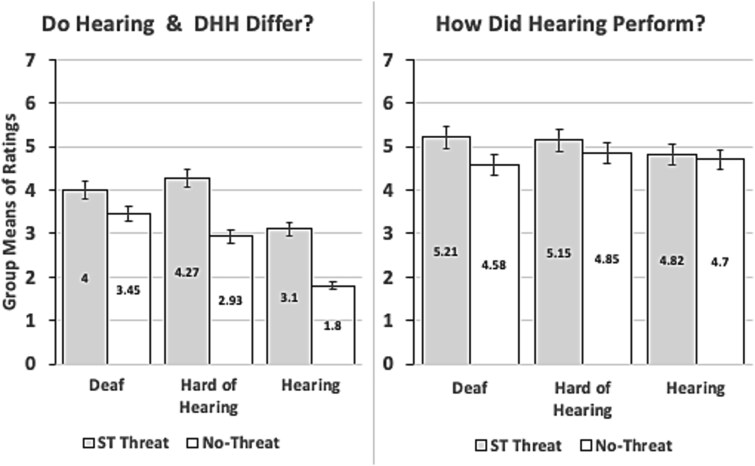
Perceptions of all participants tested under the stereotype threat (ST) and no-threat conditions on whether hearing and DHH participants differ and how the hearing participants performed. Note. Standard errors are represented in the figure by the error bars for each column.

With regard to the post-task questions (see Figure 4), the initial two questions assessed whether the stereotype threat condition activated the stereotype threat effect in that group as compared to the group receiving the instructions for the no-threat condition. The responses to the first question—“To what extent are there differences between hearing and deaf/hard-of-hearing people on these math tasks?”—showed that the response ratings for all three participant groups tested under the stereotype threat condition were significantly higher than those tested under the no-threat condition, *F*(1, 490) = 53.28, *p* < .001, η_p_^2^ = .098, midway between a medium and large effect size. There was also a significant difference for the main effect of the three groups, *F*(2, 490) = 32.21, *p* < .001, η_p_^2^ = .12, a near-medium effect size. The interaction was significant between groups and the threat/no-threat conditions, *F*(2, 490) = 3.52, *p* = .030, η_p_^2^ = .01, but with an insignificant effect size. Consistent with this finding, the responses to the second post-task-question—“How well do you think hearing students performed on these math tasks?”—showed that the response ratings of the three participant groups tested under the stereotype threat condition were significantly higher than those tested under the no-threat condition, *F*(1, 340) = 13.86, *p* < .001, η_p_^2^ = .027, a small effect size. Figure 4 illustrates the mean response ratings on post-task questions 1 and 2 for all participant groups tested under the stereotype threat condition and under the no-threat condition. These relative responses confirm that the priming instructions indeed activated stereotype threat effects for those tested under the stereotype threat condition.

**Figure 5:**
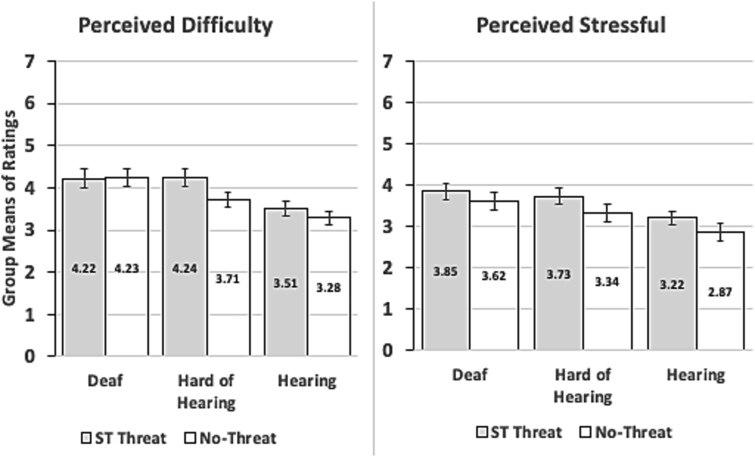
Perceptions of difficulty and stressfulness for all participant groups tested under the stereotype threat (ST) and no-threat conditions. Note. Standard errors are represented in the figure by the error bars for each column.

The final three post-task questions focused on how difficult the participants perceived the math problems to be, how stressful the math problems were to them, and how much effort they gave to solving the math problems. Figure 5 graphs the mean responses to two of the three questions comparing the participants assigned to the stereotype threat condition with those assigned to the no-threat condition. For perceived difficulty of the math problems the main effect for the three participant groups was significant, *F*(2, 490) = 17.79, *p* < .001, η_p_^2^ = .068, a medium effect size. Similarly, the main effect for the stereotype threat/no-threat condition was also statistically significant, *F*(1, 490) = 4.22, *p* = .041, η_p_^2^ = .00, an insignificant effect size. There was no significant interaction between the three participant groups and the experimental stereotype threat/no-threat condition, *F*(2, 490) = 1.86, *p* = .19. The post hoc comparisons revealed that the hearing group perceived the difficulty of the math problems significantly less than both the deaf group (*M*_diff_ = .834, *p* < .001, CI_95_ = [.503; 1.165]; *SE* = .14), Cohen’s *d* = −.628, a medium effect size, and the hard-of-hearing group (*M*_diff_ = −.579, *p* < .001, CI_95_ = [.204; .954]; *SE* = .16), Cohen’s *d* = −.436, a near-medium effect size.

For perceived stressfulness of the math problems, the main effect of the three participant groups was significant, *F*(2, 490) = 9.737, *p* < .001, η_p_^2^ = .038, a small effect size. The main effect for the stereotype threat/no-threat experimental condition was also significant, revealing that the participants under the no-threat condition were significantly less stressed, *F*(1, 490) = 5.472, *p* = .020, η_p_^2^ = .01, a negligible effect size. The post hoc comparisons showed that the hearing group was significantly less stressed than either the deaf group (*M*_diff_ = .683, *p* < .001, CI_95_ = [.316; 1.05]; *SE* = .16), Cohen’s *d* = −.464, a medium effect size, or the hard-of-hearing group (*M*_diff_ = .485, *p* = .019, CI_95_ = [.69; .901]; *SE* = .18), Cohen’s *d* = .329, a small effect size. The overall trend graphed in Figure 5 shows that the participants in the no-threat condition tended to perceive the math tasks as less difficult and less stressful, which provides validity evidence that the stereotype threat method worked.

The perceived effort given to solving the problems is not presented in Figure 5 because none of the ANOVAs revealed any significant differences. All of the participant groups perceived their effort given to solving the math tasks similarly high regardless of the stereotype threat/no-threat test condition: deaf (*M* = 5.3; *SD* = 5.1); hard of hearing (*M* = 5.4; *SD* = 5.2); and hearing (*M* = 5.07; *SD* = 5.0). This indicates that the participants in all three groups perceived themselves to have given equivalent effort to solving the math problems.

## Discussion

Stereotype threat is real and deaf and hard-of-hearing students are not immune to it. The results of this study supported the alternative hypothesis: The deaf and hard-of-hearing participants tested in the stereotype threat condition significantly underperformed on the math tests relative to the deaf and hard-of-hearing participants in the no-threat test condition. Additionally, the deaf and hard-of-hearing stereotype threat participants’ responses, compared to those tested under the no-threat condition, to the post-task experimental manipulation check questions and their perceptions of the test’s difficulty and stressfulness validated that the threat condition of the experiment worked. In other words, responses to the first two post-task questions confirmed that the instructions for the test conditions (stereotype threat and no-threat) were understood and internalized by the participants. Stereotype threats can affect performance only if a participant believes the threat is related to their social identity (Jamieson & Harkins, 2011; Quinn et al., 2010; Wout et al., 2008) and this study’s participants tested under the stereotype threat condition feared confirming the stereotype which provides evidence that such stereotypes exist. This study provided the first empirical evidence that deaf and hard-of-hearing learners are susceptible to stereotype threat, which provides support for the social model of disability.

The study’s findings contribute to the literature supporting the Minority Stress Theory (Meyer, 2003) by illustrating that DHH individuals are susceptible to stereotype threat. The significant incongruency between the participants’ self-identification as deaf or hard of hearing and their medical diagnosis (hearing ranges on audiograms) also supports that these circumstances are social constructs like other social identities of gender, ethnicity, and race. This is important because many who study or work with DHH children and adults often view any underperformance by this community to be attributed to medical differences, not social factors.

### Multiple marginalized identities and intersectional minority stress

Individuals with multiple marginalized or stigmatized identities experience greater minority stress than individuals who have one underprivileged identity (Bowleg, 2008, 2012; Crenshaw, 1991; Cyrus, 2017; Rubin-McGregor et al., 2025) and have worse mental and physical health outcomes and higher rates of substance abuse disorders (Ramirez & Pez Gulupo, 2019; Swann et al., 2020; Vargas et al., 2020). The additive multiple marginalization approach (Calabrese et al., 2015; Harari & Lee, 2021) postulates that people contending with multiple marginalized social identities experience discrimination on the basis of each of their identities. The main hypotheses of the present study did not focus on multiple marginalized identities, but the random assignment to the two experimental conditions resulted in an approximately equal distribution of gender for the DHH participants (female: 89 tested under the stereotype threat condition, 90 tested under the non-threat condition; male participants: 86 tested under the stereotype threat condition, 79 under the non-threat condition) and for hearing participants (female participants: 34 tested under the stereotype threat condition, 37 tested under the non-threat condition; male participants: 41 tested under the stereotype threat condition, 40 under the non-threat condition). This balanced distribution allowed further exploration of gender effects on math performance. This stereotype threat experiment did not specifically introduce a gender-based threat (e.g., DHH women perform worse than DHH men and hearing woman) but only a DHH-related threat. Nevertheless, the exploratory results revealed that female participants consistently underperformed compared to males within all participant groups. These results additionally demonstrated that being DHH and female invites double stereotype threat jeopardy, impacting math performance more than either identity alone.

The exploratory analyses of the impacts of ethnicity and race on math performance was problematic because the DHH samples were more ethnically and racially diverse than the hearing sample. The impacts of ethnicity and race on math performance could only be examined with the combined DHH grouping, with the findings revealing that the African American/Black DHH participants significantly underperformed compared to the White DHH participants. Since the primary focus of this study was to prime the deaf and hard-of-hearing participants on the negative stereotype that “deaf and hard-of-hearing students do not perform as well as hearing students on these types of math tasks,” no recruitment procedure was incorporated to assure sufficient sample size for the various ethnicity and race populations. Nevertheless, the African American/Black DHH participants had a greater response to the threat condition than the other DHH groups. This exploratory finding provides support for the notion that having more than one marginalized and stigmatized identity can have additional effects on minority stress.

Stapleton (2016) found that Black DHH individuals often feel *invisible* in the college classroom and described this as a common racial and audism microaggression experienced by this intersectionally marginalized group. Stapleton explained that “faculty’s inability to speak to or educate from a multicultural place leaves students feeling invisible and continues to dishonor intersectional experiences” (p. 159). *Trivialization* is another theme Stapleton identified that Black DHH individuals experience where hearing and/or White faculty do not recognize their own privilege, nor do they realize the importance for Black DHH individuals to make choices for themselves. Faculty with privilege often believe they know better than underprivileged groups about what is best for Black and/or DHH students and do not foster the students’ self-determination and self-management skills. In addition to *invisibility* and *trivialization*, Stapleton found that DHH black students face *distorted expectations* from hearing and/or White faculty because they are not aware of what makes White or hearing students privileged and Black DHH students underprivileged in a way that they can appropriately scaffold their learning. Although Stapleton did not focus on gender differences, it is possible that female students, especially female DHH students, have similar experiences of sexist and audist microagressions, biases, and oppressions.

### Practical implications

Stereotype threat is only one of a number of factors that undermine DHH people’s confidence and academic performance throughout their school years. As noted previously, more than 50% of DHH students entering college ranked below the 50th percentile in confidence in their mathematical and science abilities (Albertini et al., 2012). Lacking confidence in a skill could increase one’s susceptibility to stereotype threat (Deemer et al., 2014). One of the most effective ways to gain confidence in mathematical skills is to experience good math instruction and extensive practice of one’s mathematical skills, especially math word problems. However, it is questionable if many DHH students receive good math instruction because the majority of instructors teaching mathematics and word problem solving to DHH students in grades 6–12 lack adequate preparation and certification in mathematics to teach those skills (Kelly et al., 2003). Lacking confidence in mathematical skills and the ability to resist related stereotype threats may have potentially lasting effects that could influence their career advancement because of internalized stereotypes and continuing bias of their hearing colleagues and supervisors in their work environment.

Kelly et al. (2015) demonstrated the persistent difficulty that DHH alumni aged 25–59 with 4-year bachelor degrees have in achieving promotions and career advancement compared to their similarly aged hearing peers from the same university. Based on a survey of 1,196 DHH and 940 hearing alumni, the hearing alumni had a significantly greater probability of being promoted throughout their careers; they were 3.2 times more likely to advance to mid-level manager and 5.7 times more likely to advance to senior manager (Kelly et al., 2015, 2016, p. 475). In a broader context, analyzing 2010 census data for the total U.S. labor force shows that only one tenth of one percent (.001) of DHH people held mid-level management positions compared to 7.7% of hearing people, and only two-hundredths of 1% (.0002) of DHH people were categorized as general operational managers or chief executives compared to 1.39% of hearing people (Kelly et al., 2016, p. 476). Both DHH and hearing alumni who were promoted to management positions expressed the highest satisfaction with their current jobs and careers (Kelly et al., 2015, 2016). One can only assume that the continuing challenges to job promotion and career advancement for DHH people are due, in part, to internalized stereotypes, biases, misconceptions, and other types of oppression experienced throughout DHH individuals’ education, some of which undoubtedly exist in work environments that are dominated by hearing people and hearing culture.

### Limitations of study

It was not possible to obtain any standardized test scores at entry to college for the hearing peer group in view of the university’s security and confidentiality policies. Thus, there was no way to compare the deaf and hard-of-hearing students’ math ability levels at entry to college with the math ability levels of their hearing peers to ascertain whether the groups were comparable in math skills. Nevertheless, it is a reasonable assumption that the hearing student participants entered college better prepared and more confident about math than the DHH participants, given the documented challenges in mathematics that DHH students face across the educational spectrum (Blatto-Vallee et al., 2007; Kelly, 2008; Qi & Mitchell, 2012). In the present study, neither group—deaf or hard of hearing—performed equivalently to their hearing college peers. While stereotype threat effects may contribute to some of the difference in math test performance between DHH and hearing college students, it clearly does not explain such a large difference. Follow-up research should focus on how to tease apart the impacts of insufficient math learning and stereotype threat. Additionally, it is important to note that since the data for this study were collected, several well-designed research studies (Flore et al., 2018; Pennington et al., 2019) were unable to replicate stereotype threat effects on hearing female participants’ math performance. These findings support taking a cautionary approach to generalizing stereotype threat math effects to female students.

## Conclusion

The self-assigned social identities of deaf and hard-of-hearing participants tested under the stereotype threat condition resulted in significant underperformance on the math problems compared to their counterparts tested under the no-threat condition. This finding demonstrates that stereotype threat effects can degrade the math test performance of even the best academically prepared deaf and hard-of-hearing students. It does not suggest that mitigating stereotype threat alone will eliminate the mean score math gaps between DHH students and their hearing peers (see Sackett & Ryan, 2012; Tomeh & Sackett, 2022). The exploratory findings also illustrated that being a woman or being Black/African American can have additive intersectional effects and increase the risk that DHH students might experience stereotype effects. In view of these findings, educators should try to minimize stereotype threat by being conscious and cautious in the messages they convey to their students about their math abilities. This caution is supported by the research of Seo and Lee (2021), who found that stereotype threat in high school is linked to teachers’ fixed mindsets about students’ abilities. Importantly, teachers of DHH students need to discuss deaf-related oppression, including audism, sexism, and racism, and the effects found in this study in order to help their students build resilience toward these societal influences. Broader understanding of social constructs and the potential educational effects of stereotyping is essential to efforts to mitigate the compounding negative consequences of stereotype threat and other biases and forms of oppression that exist throughout the school years and even into the adult work environment.

## Supplementary Material

Appendix_enaf088

## References

[ref1] Albertini, J. A., Kelly, R. R., & Matchett, M. K. (2012). Personal factors that influence deaf college students’ academic success. Journal of Deaf Studies and Deaf Education, 17, 85–101. 10.1093/deafed/enr016. 21558157

[ref2] Blatto-Vallee, G., Kelly, R. R., Gaustad, M. G., Porter, J., & Fonzi, J. (2007). Visual-spatial representation in mathematical problem solving by deaf and hearing students. Journal of Deaf Studies and Deaf Education, 12, 432–448. 10.1093/deafed/enm022. 17548803

[ref3] Bowleg, L. (2008). When Black+ lesbian+ woman≠ Black lesbian woman: The methodological challenges of qualitative and quantitative intersectionality research. Sex Roles, 59, 312–325. 10.1007/s11199-008-9400-z.

[ref4] Bowleg, L. (2012). The problem with the phrase women and minorities: Intersectionality—An important theoretical framework for public health. American Journal of Public Health, 102, 1267–1273. 10.2105/AJPH.2012.300750. 22594719 PMC3477987

[ref5] Brown, R. P., & Pinel, E. C. (2003). Stigma on my mind: Individual differences in the experience of stereotype threat. Journal of Experimental Social Psychology, 39, 626–633. 10.1016/S0022-1031(03)00039-8.

[ref6] Calabrese, S. K., Meyer, I. H., Overstreet, N. M., Haile, R., & Hansen, N. B. (2015). Exploring discrimination and mental health disparities faced by Black sexual minority women using a minority stress framework. Psychology of Women Quarterly, 39, 287–304. 10.1177/0361684314560730. 26424904 PMC4584150

[ref7] Cohen, J. (1977). Statistical power analysis for the behavioral sciences ((revised ed.). ed.). Academic Press.

[ref8] Crenshaw, K. (1991). Mapping the margins: Identity politics, intersectionality, and violence against women. Stanford Law Review, 43, 1241–1299. 10.2307/1229039.

[ref9] Cyrus, K. (2017). Multiple minorities as multiply marginalized: Applying the minority stress theory to LGBTQ people of color. Journal of Gay & Lesbian Mental Health, 21, 194–202. 10.1080/19359705.2017.1320739.

[ref10] Deemer, E. D., Thoman, D. B., Chase, J. P., & Smith, J. L. (2014). Feeling the threat: Stereotype threat as a contextual barrier to women’s science career choice intentions. Journal of Career Development, 41, 141–158. 10.1177/0894845313483003.

[ref11] Desombre, C., Souad, A., & Delelis, G. (2017). Stereotype threat among students with disabilities: The importance of the evaluative context on their cognitive performance. European Journal of Psychology of Education, 33, 201–214. 10.1007/s10212-016-0327-4.

[ref12] Drače, S., Dolarević, V., & Šašić, E. (2025). How stereotype threat influences cognitive performance: It all depends on how you feel. International Review of Social Psychology, 38, 1–13. 10.5334/irsp.976. 40948594 PMC12372774

[ref13] Eckert, R. C., & Rowley, A. J. (2013). Audism: A theory and practice of audiocentric privilege. Humanity and Society, 37, 101–130. 10.1177/0160597613481731.

[ref14] Educational Testing Services (2024). Math review for the GRE general test quantitative reasoning measure, pp 1–192. https://www.ets.org/pdfs/gre/gre-math-review.pdf?utm_campaign=GRE%20Math%20Review%20email&utm_medium=email&_hsenc=p2ANqtz-83v7ce3pMeUUGyxHnno4wgJgpdHZNKCNJUexfifiuklCOsS8qRpdq3njaGQU-4KRPAb8mkm4Imsb5yvuvNTjHPGMraIQ&_hsmi=330557556&utm_source=HubSpot.

[ref15] Elliot, A. J., & Murayama, K. (2008). On the measurement of achievement goals: Critique, illustration, and application. Journal of Educational Psychology, 100, 613–628. 10.1037/0022-0663.100.3.613.

[ref16] Flore, P. C., Mulder, J., & Wicherts, J. M. (2018). The influence of gender stereotype threat on mathematics test scores of Dutch high school students: A registered report. Comprehensive Results in Social Psychology, 3, 140–174. 10.1080/23743603.2018.1559647.

[ref17] Gentile, A., Boca, S., & Giammusso, I. (2018). “You play like a woman!” Effects of gender stereotype threat on woman’s performance in physical and sport activities: A meta-analysis. Psychology of Sport & Exercise, 39, 95–103. 10.1016/j.psychsport.2018.07.013.

[ref18] Gonzales, P. M., Blanton, H., & Williams, K. J. (2002). The effects of stereotype threat and double-minority status on the test performance of Latino women. Personality and Social Psychology Bulletin, 28, 659–670. 10.1177/0146167202288010.

[ref19] Harari, L., & Lee, C. (2021). Intersectionality in quantitative health disparities research: A systematic review of challenges and limitations in empirical studies. Social Science & Medicine, 277, 113876. 10.1016/j.socscimed.2021.113876. 33866085 PMC8119321

[ref20] Hauser, P. C., O’Hearn, A., McKee, M., Steider, A., & Thew, D. (2010). Deaf epistemology: Deafhood and deafness. American Annals of the Deaf, 154, 486–492. 10.1353/aad.0.0120. 20415284

[ref21] Hopko, D. R., Mahadevn, R., Bare, R. L., & Hunt, M. K. (2003). The abbreviated math anxiety scale (AMAS): Construction, validity, and reliability. Assessment, 10, 178–182. 10.1177/1073191103010002008. 12801189

[ref22] Hoy-Ellis, C. P. (2023). Minority stress and mental health: A review of the literature. Journal of Homosexuality, 70, 806–830. 10.1080/00918369.2021.2004794. 34812698

[ref23] Inzlicht, M., & Schmader, T. (2012). Introduction. In M. Inszlicht & T. Schmader (Eds.), Stereotype threat: Theory, process, and application (pp. 3–14). Oxford University Press. 10.1093/acprof:oso/9780199732449.001.0001.

[ref24] Jamieson, J. P., & Harkins, S. G. (2009). The effect of stereotype threat on the solving of quantitative GRE problems: A mere effort interpretation. Personality and Social Psychology Bulletin, 35, 1301–1314. 10.1177/0146167209335165. 19407004

[ref25] Jamieson, J. P., & Harkins, S. G. (2011). The intervening task method: Implications for measuring mediation. Personality and Social Psychology Bulletin, 37, 652–661. 10.1177/0146167211399776. 21393614

[ref26] Jamieson, J. P., Peters, B. J., Greenwood, E. J., & Altose, A. J. (2016). Reappraising stress arousal improves performance and reduces evaluation anxiety in classroom exam situations. Social Psychological and Personality Science, 7, 579–587. 10.1177/1948550616644656.

[ref27] Kelly, R. R. (2008). Deaf learners and mathematical problem solving. In M. Marschark & P. Hauser (Eds.), Deaf cognition: Foundations and outcomes (pp. 226–249). Oxford University Press. 10.1093/acprof:oso/9780195368673.003.0008.

[ref28] Kelly, R. R., Lang, H. G., & Pagliaro, C. M. (2003). Mathematics word problem solving for deaf students: A survey of practices in grades 6-12. Journal of Deaf Studies and Deaf Education, 8, 104–119. 10.1093/deafed/eng007. 15448061

[ref29] Kelly, R. R., Quagliata, A. B., DeMartino, R., & Perotti, V. (2015). Deaf workers: Educated and employed, but limited in career growth. In Proceedings of the 22nd International Congress on Education of the Deaf. Athens, Greece.

[ref30] Kelly, R. R., Quagliata, A. B., DeMartino, R., & Perotti, V. (2016). 21^st^ century deaf workers: Going beyond just employed to career growth and entrepreneurship. In M. Marschark, V. Lampropoulou & E. K. Skordilis (Eds.), Diversity in deaf education (pp. 473–505). Oxford University Press. 10.1093/acprof:oso/9780190493073.003.0017.

[ref31] Kurz, K. B., Hauser, P. C., & Listman, J. D. (2016). Work-related resilience: Deaf professionals’ perspectives. JADARA, 50, 88–109. https://repository.wcsu.edu/jadara/vol50/iss3/1.

[ref32] Leigh, I. W. (2009). A lens on deaf identities. (Perspectives on deafness) 1st Edition, 223 pages. Oxford University Press. 10.1093/acprof:oso/9780195320664.001.0001.

[ref33] Leigh, I. W. (2020). Deaf identities: A maturity framework. In I. W. Leigh & C. A. O’Brien (Eds.), Deaf identities: Exploring new frontiers (pp. 1–26). Oxford University Press. 10.1093/oso/9780190887599.003.0001.

[ref34] Levy, S. R., Stroessner, S. J., & Dweck, C. S. (1998). Stereotype formation and endorsement: The role of implicit theories. Journal of Personality and Social Psychology, 74, 1421–1436. 10.1037/0022-3514.74.6.1421.

[ref35] Mauldin, L. (2020). Lessons learned: How studying cochlear implantation reveals the context in which d/Deaf identities are formed. In I. W. Leigh & C. A. O’Brien (Eds.), Deaf identities: Exploring new frontiers (pp. 96–119). Oxford University Press. 10.1093/oso/9780190887599.003.0001.

[ref36] May, A. L., & Stone, C. A. (2014). An initial investigation into the role of stereotype threat in the test performance of college students with learning disabilities. Journal of Postsecondary Education and Disability, 27, 89–106. https://www.ahead.org/professional-resources/publications/jped.

[ref37] Meyer, I. H. (2003). Prejudice, social stress, and mental health in lesbian, gay, and bisexual populations: Conceptual issues and research evidence. Psychological Bulletin, 129, 674–697. 10.1037/0033-2909.129.5.674. 12956539 PMC2072932

[ref38] Mitra, S. (2006). The capability approach and disability. Journal of Disability Policy Studies, 16, 236–247. 10.1177/10442073060160040501.

[ref39] O’Connell, N. (2022). “Opportunity blocked”: Deaf people, employment and the sociology of audism. Humanity and Society, 46, 336–358. 10.1177/0160597621995505.

[ref40] Pennington, C. R., Litchfield, D., McLatchie, N., & Heim, D. (2019). Stereotype threat may not impact women’s inhibitory control or mathematical performance: Providing support for the null hypothesis. European Journal of Social Psychology, 49, 717–734. 10.1002/ejsp.2540.

[ref41] Punch, R., Creed, P. A., & Hyde, M. B. (2006). Career barriers perceived by hard-of-hearing adolescents: Implications for practice from a mixed-methods study. Journal of Deaf Studies and Deaf Education, 11, 224–237. 10.1093/deafed/enj023. 16410608

[ref42] Qi, S., & Mitchell, R. E. (2012). Large-scale academic achievement testing of deaf and hard-of-hearing students: Past, present, and future. Journal of Deaf Studies and Deaf Education, 17, 1–18. 10.1093/deafed/enr028. 21712463

[ref43] Quinn, D. M., Kallen, R. W., & Spencer, S. J. (2010). Stereotype threat. In J. F. Dovidio, M. Hewstone, P. Glick & V. M. Esses (Eds.), The Sage handbook of prejudice, stereotyping and discrimination (pp. 379–394). SAGE Publications. 10.4135/9781446200919.n23.

[ref44] Ramirez, J. L., & Paz Galupo, M. (2019). Multiple minority stress: The role of proximal and distal stress on mental health outcomes among lesbian, gay, and bisexual people of color. Journal of Gay & Lesbian Mental Health, 23, 145–167. 10.1080/19359705.2019.1568946.

[ref45] Rohmer, O., & Louvet, E. (2009). Describing persons with disability: Salience of disability, gender, and ethnicity. Rehabilitation Psychology, 54, 76–82. 10.1037/a0014445. 19618706

[ref46] Rohmer, O., & Louvet, E. (2011). Stereotype content of disability subgroups – Testing predictions of the fundamental dimensions of social judgment. L’Année Psychologique, 111, 69–86. 10.4074/S0003503311001035.

[ref47] Rubin-McGregor, J., Heitt, J., & Hunger, J. (2025). Multiple marginalized identities, minority stress, and mental health. Social and Personality Psychology Compass, 19, e70077. 10.1111/spc3.70077.

[ref48] Sackett, P. R., & Ryan, A. M. (2012). Concerns about generalizing stereotype threat research findings to operational high-stakes testing. In M. Inszlicht & T. Schmader (Eds.), Stereotype threat: Theory, process, and application (pp. 249–263). Oxford University Press. 10.1093/acprof:oso/9780199732449.001.0001.

[ref49] Schmader, T. (2002). Gender identification moderates stereotype threat effects on women’s math performance. Journal of Experimental Social Psychology, 38, 194–201. 10.1006/jesp.2001.1500.

[ref50] Seo, E., & Lee, Y. (2021). Sterotype threat in high school classrooms: How it links to teacher mindset climate, mathematics anxiety, and achievement. Journal of Youth and Adolescence, 50, 1410–1423. 10.1007/s10964-021-01435-x. 33913043 PMC8222175

[ref51] Shakespeare, T. (2010). The social model of disability. In L. J. Davis (Ed.), The disability studies reader (pp. 266–273). Routledge.

[ref52] Silverman, A. M., & Cohen, G. L. (2014). Stereotypes as stumbling-blocks: How coping with stereotype threat affects life outcomes for people with physical disabilities. Personality and Social Psychology Bulletin OnlineFirst, 40, 1330–1340. 10.1177/0146167214542800. 25015337

[ref53] Spencer, B., & Castano, E. (2007). Social class is dead. Long live social class! Stereotype threat among low socioeconomic status individuals. Social Justice Research, 20, 418–432. 10.1007/s11211-007-0047-7.

[ref54] Spencer, S. J., Steele, C. M., & Quinn, D. M. (1999). Stereotype threat and women’s math performance. Journal of Experimental Social Psychology, 35, 4–28. 10.1006/jesp.1998.1373.

[ref55] Stapleton, L. (2016). Audism and racism: The hidden curriculum impacting Black d/Deaf college students in the classroom. The Negro Educational Review, 67, 149–168. https://www.academia.edu/35003418/Audism_and_Racism.

[ref56] Steele, C. M. (2010). Whistling Vivaldi: How stereotypes affect us and what we can do. W. W. Norton & Company. https://wwnorton.com/books/Whistling-Vivaldi/.

[ref57] Steele, C. M., & Aronson, J. (1995). Stereotype threat and the intellectual test performance of African Americans. Journal of Personality and Social Psychology, 69, 797–811. 10.1037//0022-3514.69.5.797. 7473032

[ref58] Stone, J. (2002). Battling doubt by avoiding practice: The effects of stereotype threat on self-handicapping in white athletes. Personality and Social Psychology Bulletin, 28, 1667–1678. 10.1177/014616702237648.

[ref59] Stone, J., Lynch, C. I., Sjomeling, M., & Darley, J. M. (1999). Stereotype threat effects on black and white athletic performance. Journal of Personality and Social Psychology, 77, 1213–1227. 10.1037/0022-3514.77.6.1213.

[ref60] Swann, G., Stephens, J., Newcomb, M. E., & Whitton, S. W. (2020). Effects of sexual/gender minority- and race-based enacted stigma on mental health and substance use in female assigned at birth sexual minority youth. Cultural Diversity & Ethnic Minority Psychology, 26, 239–249. 10.1037/cdp0000292. 31021146 PMC6814455

[ref61] Tomaszewski, P., Krzysztofiak, P., Kowalska, J., & Hauser, P. C. (2025). Internalized oppression and deaf people’s mental health. Scientific Reports, 15, 5268. 10.1038/s41598-025-89789-1. 39939356 PMC11822193

[ref62] Tomeh, D. H., & Sackett, P. R. (2022). On the continued misinterpretation of stereotype threat as accounting for black-white differences on cognitive tests. Personal Assessment and Decisions, 1, 1–14. 10.25035/pad.2022.01.001.

[ref63] Vargas, S. M., Huey, S. J. Jr., & Miranda, J. (2020). A critical review of current evidence on multiple types of discrimination and mental health. American Journal of Orthopsychiatry, 90, 374–390. 10.1037/ort0000441. 31999138

[ref64] Watson, D., Clark, L. A., & Tellegen, A. (1988). Development and validation of brief measures of positive and negative affect: The PANAS Scales. Journal of Personality and Social Psychology, 54, 1063–1070. 3397865 10.1037//0022-3514.54.6.1063

[ref65] Wilson, E. (2001). Foreword. In J. Davis (Ed.), Our forgotten children: Hard of hearing pupils in the schools ((3rd ed., pp. 7–8). SHHH Publications.

[ref66] Wout, D., Danso, H., Jackson, J., & Spencer, S. (2008). The many faces of stereotype threat: Group- and self-threat. Journal of Experimental Social Psychology, 44, 792–799. 10.1016/j.jesp.2007.07.005.

[ref67] Yopyk, D. J. A., & Prentice, D. A. (2005). Am I an athlete or a student? Identity salience and stereotype threat in student-athletes. Basic and Applied Social Psychology, 27, 329–336. 10.1207/s15324834basp2704_5.

