# Supplementary material for: Stereotype threat effects on deaf and hard-of-hearing college students’ mathematics performance
Source: J Deaf Stud Deaf Educ. 2026 Jan 28;31(3):435–48. doi: 10.1093/jdsade/enaf088 (PMC13343188; doi:10.1093/jdsade/enaf088)
Supplement: Appendix_enaf088 [file appendix_enaf088.docx]

**Appendix**

**Example of Arithmetic Format (add, subtract, divide, multiply)**

| 36  -11 | 25  + 9 | 12  x 5 | 56  ÷ 7 |
| --- | --- | --- | --- |

**Example of Modular Arithmetic Format**

28 Ξ (mod 13)

__ True

__ False

So 28 – 13 = 15; 15/3 = 5; Answer = True

**Example of Graduate Record Examinations “GRE-type”** **Multiple-Choice Format**

| Example 1. If the total surface area of a cube is 24, what is the volume of the cube? [answer is a] | | |
| --- | --- | --- |
| 1. 8 2. 24 3. 64 4. 48 | 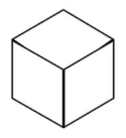 | |

Example 2. [answer is *b*]

| *n* = (7) (19^3^) | |
| --- | --- |
| Column A  The total # of positive factors of *n* | Column B  10 |
| a. The quantity in Column A is greater  b. The quantity in Column B is greater  c. The two quantities are equal  d. The relationship cannot be determined | |
